# Translation, cross-cultural adaptation, and validation of the Norwegian version of the Keratoconus Outcomes Research Questionnaire

**DOI:** 10.1186/s41687-025-00896-z

**Published:** 2025-05-22

**Authors:** Eilin Lundanes, Svein Magne Roten, Helle Kristine Falkenberg, Lena Leren, Vibeke Sundling

**Affiliations:** 1 National Centre for Optics, Vision and Eye Care, Department of Optometry, Radiography and Lighting Design, Faculty of Health and Social Sciences, University of South-Eastern Norway, Hasbergsvei 36, Kongsberg, 3616 Norway; 2 Centre for Health and Technology, Department of Nursing and Health Sciences, Faculty of Health and Social Sciences, University of South-Eastern Norway, Grønland 58, Drammen, 3045 Norway; 3 Specsavers Strømmen, Støperiveien 5, Strømmen, 2010 Norway

**Keywords:** Keratoconus outcomes research questionnaire, Translation, Cross-cultural adaptation, Validity, Reliability, Rasch analysis

## Abstract

**Purpose:**

To translate and adapt the Keratoconus Outcomes Research questionnaire (KORQ) to Norwegian language, culture, and environment, and to validate the translated version in a Norwegian population with keratoconus.

**Methods:**

KORQ was translated to Norwegian using a multi-step methodology. Persons with keratoconus submitted responses to KORQ-NO and NEI VFQ-25 through a digital platform, and a retest of KORQ-NO was performed over the telephone. Additional data from a clinical intervention study was included. The psychometric properties of KORQ-NO were assessed by Rasch analysis. Test-retest reliability, construct validity, and responsiveness were explored by Intraclass Correlation Coefficients, Spearman correlations and Wilcoxon Signed-rank test.

**Results:**

KORQ-NO and NEI VFQ-25 were completed by 165 participants with keratoconus. With few adjustments, the “Activity limitations” (AL) and “Symptoms” (S) subscales of KORQ-NO exhibit acceptable psychometric properties with good model fit, high internal reliability, and well-targeted items to the population. Deletion of four items (AL3, AL3b, AL12, AL15) improved dimensionality of the “Activity limitations” subscale. Differential item functioning was present in two items (AL4 and AL6). Participants and optometrists confirmed content validity, and KORQ-NO exhibited good test-retest reliability (AL ICC = 0.90 and S ICC = 0.81), construct validity, and responsiveness.

**Conclusions:**

Successful translation and adaptation of KORQ to Norwegian language, culture and environment was confirmed by acceptable psychometric properties, with good validity, reliability, and responsiveness. The authors support the use of KORQ-NO in research, clinical practice, and as documentation for national insurance benefit applications.

**Supplementary Information:**

The online version contains supplementary material available at 10.1186/s41687-025-00896-z.

## Abstract (plain language summary)

**Why?:**

Keratoconus is an eye disease occurring in early adolescence. Persons with keratoconus often have reduced quality-of-life (QoL) despite only subtle changes in visual functions. There is a need of a Norwegian questionnaire to measure vision-related QoL in persons with keratoconus.

**What?:**

The aim was to translate a vision-related QoL questionnaire specific for keratoconus (KORQ) into Norwegian, and to evaluate the translation.

**Main findings and so what?:**

This study has shown that the Norwegian translation of the questionnaire works as intended. The questionnaire is relevant for use in research on keratoconus, clinical practice, and for national insurance benefit applications. The Norwegian translation of the questionnaire (KORQ-NO) is freely available.

**Supplementary Information:**

The online version contains supplementary material available at 10.1186/s41687-025-00896-z.

## Background

Keratoconus is a progressive, chronic corneal disease occurring in early adolescence. Asymmetric corneal thinning and protrusion result in irregular astigmatism, distorted and reduced vision. Common symptoms include polyopia, glare, reduced night vision, photophobia, and eye strain [1, 2]. The prevalence of keratoconus worldwide ranges from 0.2 to 3300 per 100.000 depending on diagnostic criteria and geographic location [3]. In Norway the prevalence is estimated to 192 per 100.000, with an incidence of 20 per 100.000 in the general population [4].

It is well-established that keratoconus compromises vision-related quality of life (VR-QoL) [5–7]. The visual distortions and symptoms of keratoconus are unpredictable, and clinical measurements, such as visual acuity, are not likely to reflect the impact on visual function [5]. Assessment of VR-QoL is challenging as several latent traits may contribute, such as visual symptoms, ocular surface symptoms, general symptoms, emotional well-being, activity limitations, mobility, convenience, health concerns, social and economic well-being and so forth [8]. Well-functioning questionnaires, commonly referred to as patient-reported outcome measures (PROMs), may be used to assess such latent traits [8]. Many studies have used the National Eye Institute Visual Functioning Questionnaire (NEI VFQ-25), a generic vision-related PROM originally developed for age-related eye disease [9], to investigate VR-QoL in keratoconus [6, 10–13]. However, a generic PROM usually has reduced sensitivity with worse validity and responsiveness to change compared to a disease-specific PROM [8, 14].

Keratoconus Outcomes Research Questionnaire (KORQ) was the first available keratoconus-specific PROM, developed and validated by Khadka and colleges in Australian populations [15, 16]. The English version is also validated in a population in India [17]. So far, KORQ has been translated to Danish [18], Italian [19], Portuguese [20], Spanish [21] and German [22]. Most translations exhibit minor issues with multidimensionality of the questionnaire, solved by collapsing or deleting items [16, 18, 20–22]. Studies evaluating KORQ found excellent test-retest reliability [17, 19, 21] and weak to moderate positive correlations between KORQ and corrected visual acuity [17, 20, 22] and steepest corneal curvature [18, 19]. To date, no keratoconus-specific PROM is available in Norwegian.

The Norwegian National Insurance Act § 10 − 7 states that persons with keratoconus may receive insurance benefits for specialty contact lenses if visual acuity and/or visual function is significantly improved compared to conventional correction glasses or contact lenses [23, 24]. Therefore, a validated, keratoconus-specific PROM in Norwegian may not only be useful in research and clinical practice but also serve as a valuable tool for documenting improvements beyond clinical measures for the Norwegian Labour and Welfare Administration when considering insurance benefit applications.

Translation, cross-cultural adaptation, and validation in the target population is important before implementation of a PROM [25]. There is no global consensus on cross-cultural adaptation of PROMs [26], but a multistep methodology including forward- and back-translation is recommended [25]. The Consensus-based Standards for selection of health status Measurement Instruments (COSMIN) checklists can serve as a guide in validation of PROMs [27–29]. The purpose of this study was to translate and adapt KORQ to Norwegian language, culture, and environment, and to validate the translated version in a Norwegian population with keratoconus.

## Methods

### KORQ, translation and adaptation to Norwegian

KORQ comprises two subscales, the 18 items “Activity limitations” (AL) and the 11 items “Symptoms” (S). All items have a four-point rating scale ranging from 1 to 4; 1 = Not at all, 2 = A little, 3 = Quite a bit, and 4 = A lot. A “Not applicable” option, treated as missing data in analysis, is available for all items. Patient demographics are recorded on the front page of the questionnaire. The English version of KORQ is available in Appendix 1.

**Fig. 1:**
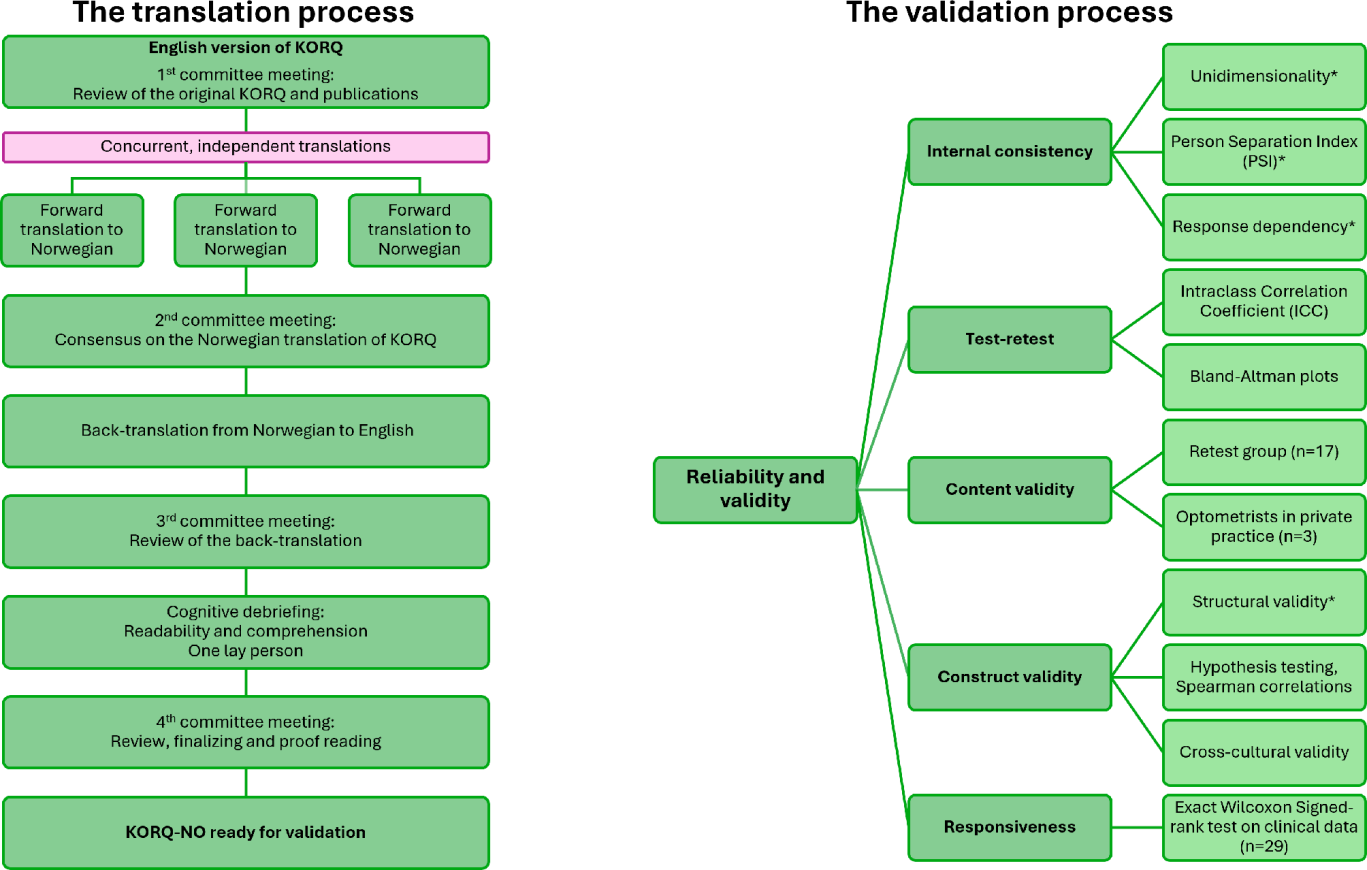
The translation and validation process. *Note:* The translation process is illustrated to the left. Norwegian is the native language of all committee members and translators (SMR, VS and HKF), and all are fluent in English. English is the native language of SG, and SG is fluent in Norwegian. The lay person doing the cognitive debriefing has no background in optometry and vision science. The validation process, in accordance with the COSMIN checklist, is illustrated to the right. For definitions and details of methods used to evaluate each property, please review appendix 2. Additionally, Rasch analysis was applied, as described in the methods section. * Properties included in the Rasch analysis. *Abbreviations:* KORQ: Keratoconus Outcomes Research Questionnaire, KORQ-NO: The Norwegian version of KORQ, SMR: Svein Magne Roten, Master of Optometry Student, VS: Vibeke Sundling, Professor, HKS: Helle Kristine Falkenberg, Professor, SG: Stuart Gilson, Associate Professor

The Norwegian translation of KORQ (KORQ-NO) was made with permission from the developers (Khadka). The translation process followed principles described in literature [25, 26, 30, 31], illustrated in Fig. 1. The translation was performed concurrent and independently by three Norwegian optometrists fluent in English (SMR, VS and HKF). Two of the translators (VS and HKF) have academic and clinical background from English-speaking countries and are experienced in translation work. Discrepancies in the translations were discussed, and consensus was reached. The Norwegian to English back translation was undertaken by a native English speaking academic fluent in Norwegian. Cognitive debriefing was undertaken with a lay person without background in optometry ensuring comprehensiveness of the content.

### Reliability and validity according to the COSMIN checklist

The Consensus-based standards for the selection of health status measurement instruments (COSMIN) has developed a consensus-based checklist to evaluate the quality of studies on measurement properties [27–29]. The COSMIN checklist was applied to assess reliability and validity of KORQ-NO. Figure 1 gives an overview of the properties assessed, and appendix 2 contains definitions and detailed descriptions of the methods used to evaluate each property. Statistical analysis included Intraclass Correlation Coefficients and Bland-Altman plots with Limits of Agreement, Spearman correlations and Exact Wilcoxon Signed-rank test. IBM Statistical Package for Social Sciences (SPSS, Inc. Chicago, IL, USA for Windows 10 (version 29)) [41] was used for statistical analysis.

### Rasch measurement theory

KORQ-NO was assessed using Rasch Measurement Theory in accordance with the development study of the original KORQ [15] and several other validation studies [16–18, 22]. Rasch measurement theory is a probabilistic, mathematical model commonly used to assess the psychometric properties of PROMs [34]. The model has three requirements for valid estimates; unidimensionality, invariance, and ordered response categories [35]. Ordinal scores are transformed to a linear logit scale, essential for creating sum scores and calculating change scores or standard deviations. Estimates of the relative difficulty of each item (item location) and the relative ability of each respondent (person location) are placed on a common logit scale [36]. With the scoring of KORQ-NO, lower person location logits relate to less impact on VR-QoL, whereas lower item location logits suggest more difficult items.

Rasch analysis were performed using RUMM 2030 + for Windows (RUMM Laboratory, Australia, version 5.8.1) [37]. The “Activity limitations” and “Symptoms” subscales of KORQ-NO were analysed independently by the polytomous partial credit model of Rasch [38]. NEI VFQ-25 was analysed by the same model. An overview of the properties included in the Rasch analysis with expected values is provided in Table 1. A large sample size is required for robust Rasch; however, the impact of sample size varies across different properties. Recommendations are included in Table 1.

**Table 1:** Overview of the Rasch analysis

Rasch analysis	Function name	Expected values & sample size recommendations
Overall model fit	Item-trait interaction	X^2^*p* > 0.05/# items confirms that the data is not statistically different from the model [38]
Reliability *Comparable to Cronbach’s α*	Person Separation Index (PSI)	> 0.85 is indicative of the ability of the scale to separate persons along the trait [34] Vaguely impacted by sample size [39]
***Unidimensionality*** *Measurement of one latent trait only*	Principal Component Analysis (PCA)	Proportion of statistically significant *t*-tests < 5% [34] Eigenvalue of the first principal component < 2.0–3.0 [15, 16, 18]
Response dependency	Correlations of item residuals	Correlations 0.2 above the mean item residuals indicate dependency, hence item redundancy [40]
***Invariance*** *Equal performance of items across various person factors or groups*	Differential Item Functioning (DIF)	Statistically significant differences among mean residuals indicate DIFs (Bonferroni-adjusted p-value < 0.05) [40]. The following person factors included:
		- Age (under/on vs. above mean age)
		- Sex (female vs. male)
		- Visual correction type (uncorrected/spectacles/soft contact lenses vs. rigid gas permeable lenses)
		- History of treatment (yes vs. no) Recommended sample size: 100 to 300 per subgroup [35]
***Ordered response categories***	Item threshold parameters Category probability curves	Well-separated and ordered thresholds [38] Recommended sample size: At least 10 responses per response category [35]
Targeting	Mean persons location Person-item threshold distribution graphs	Close to 0, when mean items location set to 0 by default, indicates good targeting to the population [40] Close alignment between persons and items along the logit scale indicates good targeting [40]
Item fit	Item fit residuals	Items with fit residuals within +/-2.50 with low X^2^ and Bonferroni adjusted *p* > 0.05 fit the model [40] Recommended sample size: >200 [35]
Person fit	Person fit residuals	Person misfit is indicated when fit residuals +/- ∞, marked as extreme [40]

Note: Requirements of the Rasch model include unidimensionality, invariance and ordered response categories

### Recruitment, data collection and ethics

Persons with keratoconus aged 18 years and older were invited to participate through a social media user group, “Keratoconus Norway”. “Nettskjema”, a digital platform provided by the University of Oslo, was utilized to distribute two self-reporting questionnaires on VR-QoL: the disease-specific KORQ-NO, and the vision-generic NEI VFQ-25 [32]. NEI VFQ-25 was included to enable assessment of construct validity of KORQ-NO. Participants were asked to leave their telephone number for a retest of KORQ-NO. Three to four weeks after initial completion of the questionnaires, the first author (EL) contacted the participants to repeat KORQ-NO and collect quantitative data to facilitate assessment of test-retest reliability and content validity. In addition, data from a clinical intervention study (Manuscript submitted for publication) were included to allow assessment of construct validity and responsiveness.

All participants provided written informed consent. The research was conducted in accordance with the Helsinki declaration [33]. Both the current study and the clinical intervention study were approved by the Norwegian Center for research data (SiKT reference number 934897 & 953377), and the intervention study was approved by the Regional Committee for Medical Research Ethics (REK 953377).

## Results

### Translation, cross-cultural and environmental adaptation

The three, concurrent, independent Norwegian translations possessed few differences, and the consensus process was straightforward. There were very few minor differences between the original version of KORQ and the English back-translation from the Norwegian translation. The translators discussed the term “computer screen” in AL1 and agreed to adjust the term to “digital devises” to account for constant changes in technology. Due to weather differences between Australia and Norway, the translators decided to add a question AL3b: *“How much does your vision interfere with driving in poor visibility (rain/snow/fog)?”*. Items such as S2 *“How much are you troubled by glare?”* and S3 *“How much does a bright sunny day interfere with your ability to see?”* were thoroughly discussed, but the items were kept because light reflections on snow during winter can be intense. The phrase *“windy days”* in item S7 was adjusted to *“a lot of wind”*, and the phrases *”dry/dusty days”* in items S9 and S10 were changed to *“dry air”* and *”a lot of dust in the air”* for better accuracy in Norwegian. The adjustments were included in the final version of KORQ-NO prior to data collection for validation.

### Demography

In total, 165 participants completed KORQ-NO and NEI VFQ-25,136 through “Nettskjema” and 29 as part of the clinical intervention study. Seventeen participants completed the retest of KORQ-NO. Table 2 presents an overview of demographics. There were no statistically significant differences in sex or age between the groups. The retest group had significantly more participants using contact lenses and fewer participants with history of surgical treatment.

**Table 2:** Demographic characteristics of the participants

**Demography**	All participants	Retest group	Clinical group	Chi-square statistics	
	*n* = 165	*n* = 17	*n* = 29	All vs. Retest	All vs. Clinical
**Sex**,** n (%)**					
Female	80 (48.5)	5 (29.4)	7 (24.1)	X^2^=0.29	X^2^=4.16
Male	85 (51.5)	12 (70.6)	22 (76.9)	*p* = 0.86	*p* = 0.13
**Age**					
Median	41	42	34	X^2^=153.00	X^2^=372.97
Range	19–72	29–61	20–68	*p* = 0.27	*p* = 0.32
**Optical correction**,** n (%)**^**a**^					
No correction	9 (5.5)	1 (5.9)	5 (17.2)	X^2^=13.03	X^2^=6.07
Spectacles	43 (26.1)	2 (11.7)	14 (48.3)	*p* = 0.04*	*p* = 0.73
Soft Contact Lenses	18 (10.9)	3 (17.6)	2 (6.9)		
Rigid Contact Lenses	95 (57.6)	11 (64.7)	8 (27.6)		
**Surgical treatment**,** n (%)**^**b**^					
No treatment	77 (46.7)	9 (52.9)	18 (62.1)	X^2^=0.94	X^2^=13.79
Corneal crosslinking (CXL)	57 (34.5)	6 (35.3)	10 (34.5)	*p* = 0.92	*p* = 0.008**
Corneal transplant (CT)	27 (16.4)	2 (11.8)	0 (0.0)		
Intracorneal ring segments	2 (1.2)	0 (0.0)	1 (3.4)		
Missing data	2 (1.2)	0 (0.0)	0 (0.0)		

Notes: Significance levels: *p < 0.05, **p < 0.01

^a^ Participants reporting the use of more than one optical correction type were counted as follows: Participants using rigid contact lenses and/or soft contact lenses and/or spectacles were placed in the rigid contact lenses group. Participants using soft contact lenses and spectacles were placed in the soft contact lenses group. Participants using spectacles only were placed in the spectacles group

^b^ Participants reporting more than one surgical treatment were counted as follows: Participants with history of corneal crosslinking and corneal transplantation were placed in the corneal transplantation group. Participants with history of corneal crosslinking only were placed in the corneal crosslinking group

### The Rasch analysis

Results from the Rasch analysis of “Activity limitations” and “Symptoms” subscales, and NEI VFQ-25, are summarized in Table 3. The Rasch analysis revealed several issues with the psychometric properties of the “Activity limitations” subscale as the overall fit to the model was poor, multidimensionality was indicated, and response dependency was evident between one item pair, AL3 *“driving at night”* and AL3b *“driving in poor visibility”* (*r* = 0.537). Most items did not differ across the person factors. However, AL 4 *“reading signs”* displayed statistically significant Differential Item Functioning (DIF) by age (*p* < 0.001) with higher scores in the older age group and AL 6 *“walking in stairs”* displayed statistically significant DIF by sex (*p* < 0.0001) with higher scores in females.

On the contrary, the psychometric properties of the “Symptoms” subscale were good. The proportions of statistically significant *t-*tests were slightly above the limit, but the eigenvalue of the first contrast was acceptable for unidimensionality. Both subscales exhibit excellent reliability with high Person Separation Index (PSI).

NEI VFQ-25 did not fulfil any of the requirements of Rasch, with presence of multidimensionality, invariance for item 9 *“Walking down steps*,* stairs or curbs in reduced lighting or darkness”*, and disordered thresholds for 12 of the items.

**Table 3:** Summary of Rasch analysis

	Overall fit		Reliability	Unidimensionality		Response dependency	Invariance	Response categories	Targeting	Item misfit	Person misfit
	Item-trait interaction		PSI	Principal Component Analysis			DIF	# items disordered	Mean persons location [SD]		
	Chi-square	*p*		% stat. sign. t-tests	Eigenvalue of 1st contrast	# item pairs				# items	# persons
Activity Limitations	64.39	0.005	0.94	12.73	2.57	1	By age: AL4^a^ By sex: AL6^a^	1 ^c^	-0.07 [1.66]	1^d^	3
Symptoms	33.92	0.05	0.85	6.67	2.08	0		0	0.35 [1.17]	0	1
NEI VFQ-25	120.00	< 0.001	0.94	18.79	3.46	6	By sex: N9^b^	12	-2.28 [1.23]	3^e^	0

Abbreviations: PSI: Person Separation Index, DIF: Differential Item Functioning, SD: standard deviation

^a^ AL4 “Reading signs”, AL6 “Walking in stairs”; ^b^ N9 “Walking down steps, stairs, or curbs in reduced lighting or darkness”; ^c^ Item with disordered thresholds: AL 15 “Doing household tasks”; ^d^ Misfitting item: AL12 “Doing your hobbies”; ^e^ Misfitting items: N1 “Overall health”, N4 “Pain or discomfort in or around the eyes”, N24 “Dependent on help from others”

### Response categories

All items demonstrated well-functioning response categories with ordered and well-spaced thresholds, except AL15 *“doing household tasks”*. Most participants (56.4%) responded “not at all” to this item, and only two participants (1.2%) responded “a lot.” Response frequency tables are available (Appendix 3).

### Targeting, item fit, and person fit

**Fig. 2:**
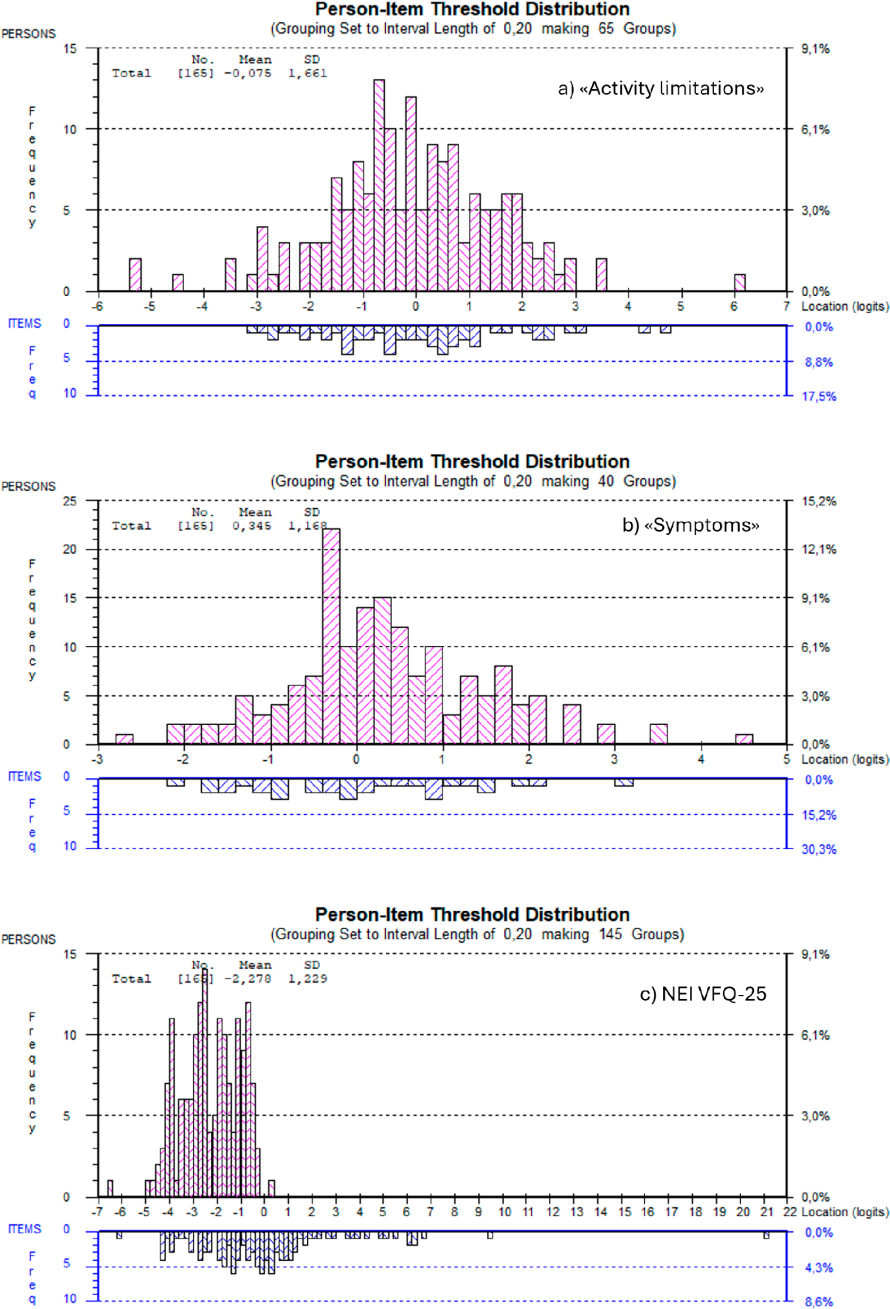
Targeting. *Note:* Person-item distribution for (**a**) “Activity limitations” (top), (**b**) “Symptoms” (middle), and (**c**) NEI VFQ-25 (bottom), illustrating superior targeting of “Activity limitations” and “Symptoms” over NEI VFQ-25. Negative logit person locations indicate less activity limitations and symptoms (better VR-QoL), whereas positive logit person locations indicate more limitations or symptoms (lower VR-QoL). Items with negative logit location indicate more demanding activities or severe symptoms, and positive logit locations indicate easier activities or less severe symptoms

The mean person locations confirm good targeting to the study population for both subscales and poor targeting for NEI VFQ-25. Only one item, AL12 *“doing your hobbies”*, displayed individual item misfit (fit residual = 3.667, *p* = 0.04). Figure 2 illustrates how the KORQ-NO items and persons fit well along the logit scale with very few outliers, whereas several items of NEI VFQ-25 do not fit the population.

### Iterative Rasch analysis for improvement of psychometric properties

To improve overall fit and dimensionality of the “Activity limitations” subscale, items were deleted one by one followed by iterative Rasch analysis. First, item AL3b *“driving in poor visibility”* was removed due to the response dependency. Thereafter, AL3 *“driving at night” was removed as r*esponse dependency appeared between AL2 *“driving in daylight”* and AL3. Due to item misfit and disordered response thresholds respectively, items AL12 *“doing your hobbies”* and AL15 *“doing household tasks”* were removed. Deletion resulted in better overall fit as well as improvements in unidimensionality and response dependency. Table 4 presents a summary of statistics after removal of items.

**Table 4:** Summary of results from iterative Rasch analysis of “activity limitations”

	Overall fit		Reliability	Unidimensionality		Response dependency	Invariance	Response categories	Targeting	Item misfit	Person misfit
	Item-trait interaction		PSI	Principal Component Analysis			DIF	# items disordered	Mean persons location [SD]		
	Chi-square	*P* ^a^		% stat. sign. t-tests	Eigenvalue of 1st contrast	# item pairs				# items	# persons
Initial AL subscale	64.39	0.00003	0.94	12.72	2.57	1	By age: AL4 By sex: AL6	1	-0.07 [1.66]	1	3
Remove AL3b	49.78	0.003*	0.94	13.33	2.41	1	No change	1	-0.13 [1.63]	1	3
Remove AL 3	40.59	0.012*	0.93	13.94	2.37	0	No change	1	-0.20 [1.60]	1	3
Remove AL12	39.05	0.011*	0.93	7.27	2.38	0	No change	1	-0.18 [1.67]	0	4
Remove AL15	33.17	0.021*	0.93	2.42	2.27	0	No change	0	-0.06 [1.68]	0	4
Ideal values		> 0.05/ #items	> 0.85	< 5%	< 2.00 to < 3.00	0		0	Close to 0	0	0

Note: *not statistically significant, indicate acceptable overall fit to the Rasch model

^a^Bonferroni corrected p-values. Ideal values: 19 items = > 0.0026, 18 items = > 0.0028, 17 items = > 0.0029, 16 items = > 0.0031, 15 items = > 0.0033

### The COSMIN checklist

#### Reliability; internal consistency, test-retest reliability, and measurement error

**Fig. 3:**
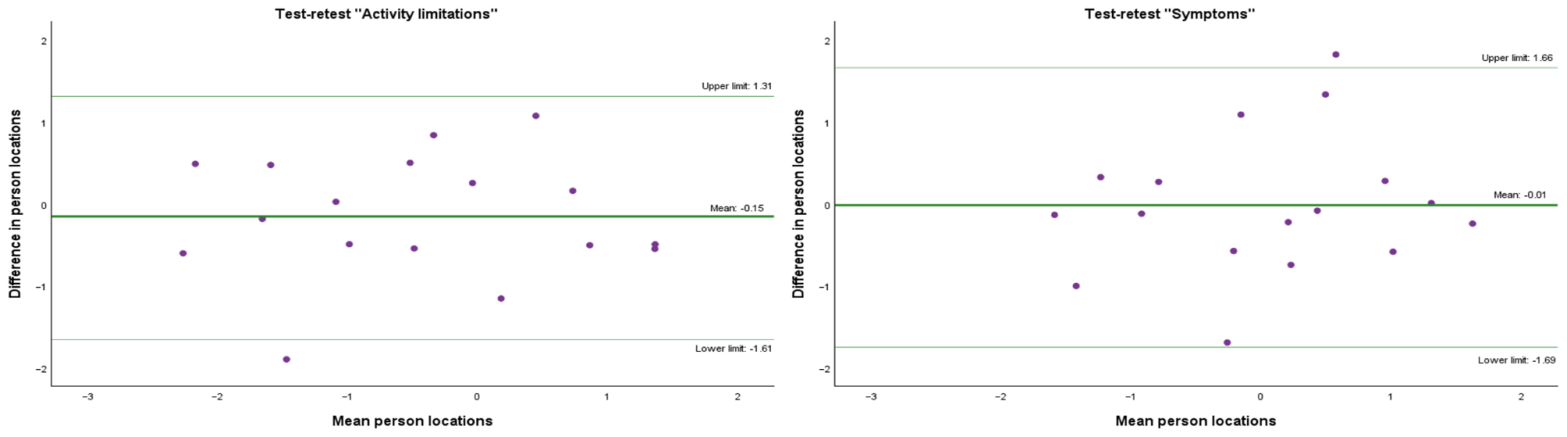
Test-retest reliability illustrated by Bland-Altman plots. *Note:* Bland-Altman plots for “Activity limitations” (right) and “Symptoms” (left) show the mean differences between the test and retest results (light green) and the upper and lower limits of agreement (dark green). Each participant is represented by a purple dot. As most participants are within the upper and lower limits of agreement, with no linear correlation, acceptable test-retest reliability is confirmed

The above-mentioned results from the Rasch analysis support acceptable levels of internal consistency of KORQ-NO. “Activity limitations” ICC = 0.90 (95% confidence intervals 0.73–0.96) indicates good to excellent test-retest reliability. “Symptoms” ICC = 0.81 (95% confidence intervals 0.47–0.93) indicates low test-retest reliability due to the large confidence intervals. The Bland-Altman plots (Fig. 3) illustrate acceptable limits of agreement between the test and retest person locations for both “Activity limitations” (linear regression coefficient = 0.08, t = 0.046, *p* = 0.964) and “Symptoms” (linear regression coefficient = 0.154, t = 0.679, *p* = 0.508).

### Content and construct validity

The seventeen retest participants confirmed relevance of most items of KORQ-NO. One participant articulated that *“this questionnaire describes my life perfectly”*. Both female and male participants perceived the item AL15 *“doing household tasks (e.g. cleaning*,* ironing*,* washing*,* washing up)”* as irrelevant, claiming their vision did not affect the ability to perform such tasks. In the back-translation from Norwegian to English the same item was phrased *“to do housework (e.g. cleaning*,* ironing*,* washing”)*, closely resembling the original item. Itching, dizziness, nausea, and exhaustion were symptoms participants mentioned as important to them but lacking in KORQ-NO. Face validity was questioned as the questionnaire does not include instructions. Participants using more than one type of optical correction would answer the questions differently depending on correction type. Fluctuations in symptoms are not covered by KORQ-NO, and several participants expressed uncertainty whether to respond based on their best day, worst day, or something in between.

The experienced optometrists’ comments pointed to irrelevance of item AL6 *“walking up/down steps”* and AL7 *“walking onto things”* as these issues are rarely reported in the clinic. One of the optometrists thought AL18 *“use of a camera*,* microscope*,* binoculars etc.”* was confusing, as it could be related to low-vision aids, not commonly used by persons with keratoconus. Except item S4 “*How much are you troubled by wearing rigid gas permeable contact lenses?*”, KORQ-NO does not include contact lens specific items. This was seen as a strength by two optometrists, whereas the third optometrist argued that the questionnaire should cover more contact lens specific aspects.

Construct validity, including structural and cross-cultural validity was confirmed by the abovementioned results of the Rasch analysis. Spearman correlations between the RUMM2030 + calculated person locations and the person locations given by the ready-made spreadsheets by the KORQ developers [15] were close to perfect for both “Activity limitations” (r_s_= 0.998, *p* < 0.001) and “Symptoms” (r_s_= 0.994, *p* < 0.001), suggesting excellent cross-cultural validity. Also, Spearman correlations suggest strong negative correlations between “Activity limitations” person locations and NEI VFQ-25 sum scores (r_s_= -0.844, *p* < 0.001) and “Symptoms” person locations and NEI VFQ-25 sum scores (r_s_= -0.714, *p* < 0.001).

Calculations of Spearman correlations between person locations for both subscales and four clinical measurements at baseline, indicate no statistically significant correlations, except a weak correlation between “Activity limitations” and near visual acuity (Table 5).

**Table 5:** Spearman correlations between KORQ-NO and clinical measurements

Spearman correlations	Binocular DVA *r*_s_ (*p*)	Binocular NVA *r*_s_ (*p*)	Asymmetry *r*_s_ (*p*)	Binocular CS *r*_s_ (*p*)
Activity limitations	0.091(0.635)	0.368 (0.05)*	0.088 (0.649)	-0.235 (0.221)
Symptoms	-0.153 (0.429)	0.193 (0.316)	-0.161(0.405)	0.050 (0.796)

Note: Spearman correlations show no statistically significant correlations between person locations on “Activity limitations” and “Symptoms” subscales and clinical measurements, except between “Activity limitations” and near visual acuity

**Fig. 4:**
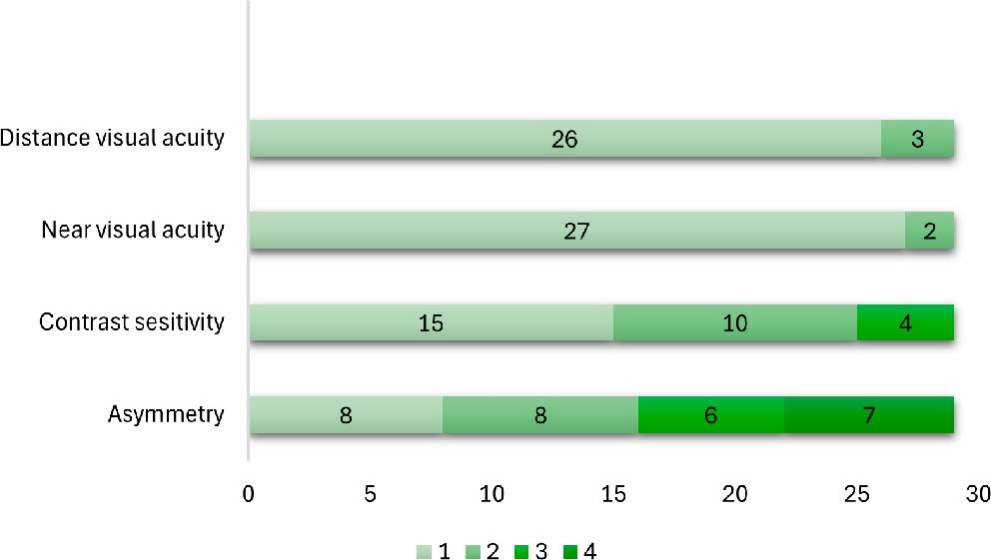
Distribution of participants on clinical measurements. *Note:* Group numbers relate to the following classifications: Distance and near visual acuity (logMAR): Group 1 = No vision impairment (VA < 0.00-0.28), Group 2 = Mild vision impairment (VA 0.30–0.48), Group 3 = Moderate vision impairment (VA 0.50–1.28) and Group 4 = Blindness (VA > 1.30). Contrast sensitivity: Group 1 = Normal (CS 1.92–1.72), Group 2 = Normal Age 60+ (CS 1.67–1.52), Group 3 = Moderate reduction (CS 1.48–1.04), Group 4 = Severe reduction (CS 0.52-1.00). Asymmetry: Group 1 = < 1 line, Group 2 = 1–2 lines, Group 3 = 2–3 lines, Group 4 = > 3 lines

Most participants in the clinical study had no visual impairment and normal contrast sensitivity, indicative of mild stages of keratoconus. Figure 4 illustrates the distribution of participants in four groups for distance and near visual acuity, contrast sensitivity and asymmetry.

**Fig. 5:**
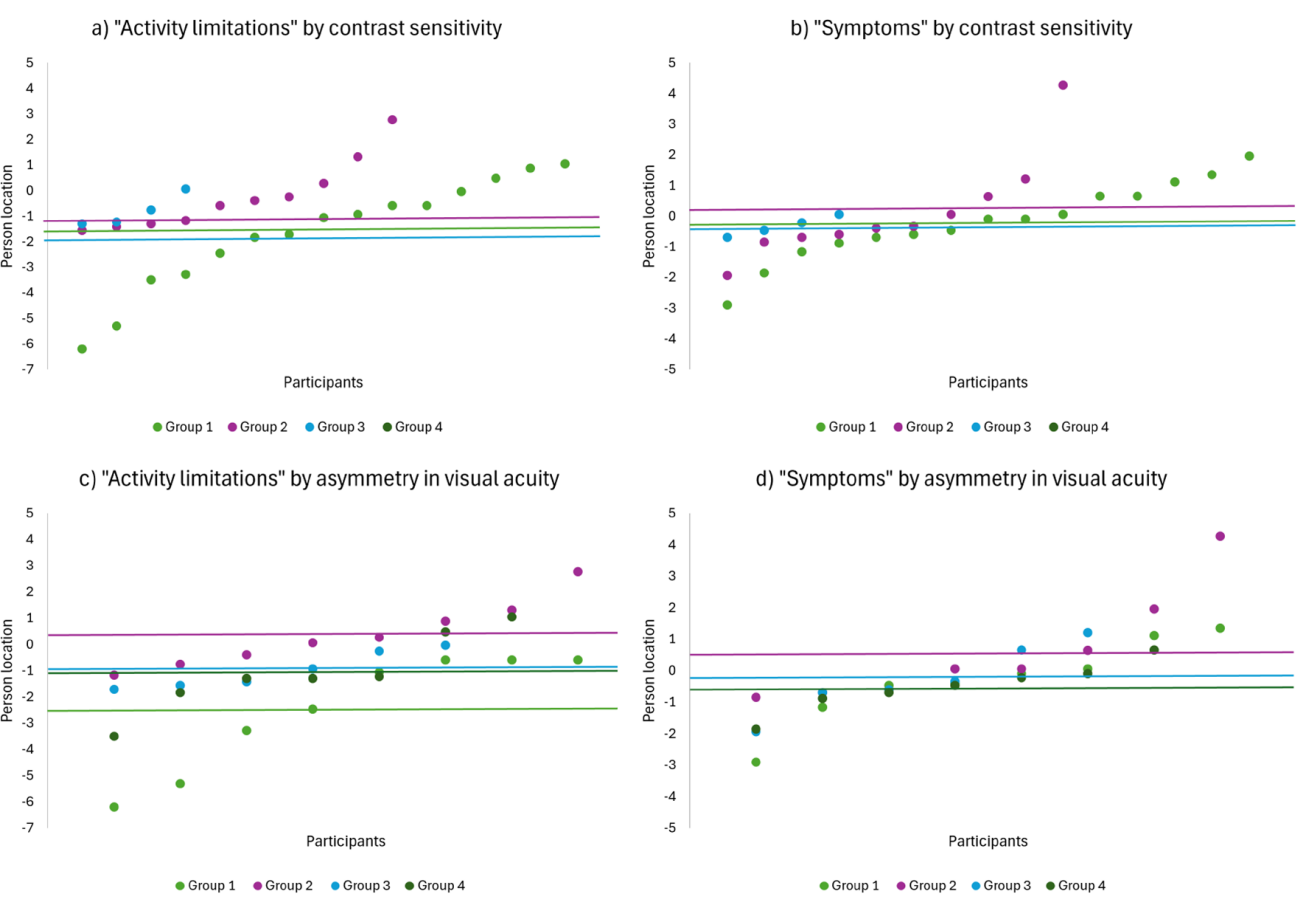
Distribution of person locations by groups for contrast sensitivity and asymmetry in visual acuity. *Note:* The y-axis presents the person locations. Each dot represents one participant. The lines indicate the mean of each group. The graphs suggest that persons with normal contrast sensitivity have lower person locations (better VR-QoL) on both “Activity limitations” and “Symptoms” subscales, whereas persons with mild asymmetry in visual acuity (group 2 and 3) have higher person locations (lower VR-QoL) on both subscales. Contrast sensitivity: Group 1 = Normal (CS 1.92–1.72), Group 2 = Normal Age 60+ (CS 1.67–1.52), Group 3 = Moderate reduction (CS 1.48–1.04), Group 4 = Severe reduction (CS 0.52-1.00). Asymmetry: Group 1 = < 1 line, Group 2 = 1–2 lines, Group 3 = 2–3 lines, Group 4 = > 3 lines

There were no statistically significant differences in person locations between any of the groups for all clinical measurements (all X^2^ p-values > 0.05), except between “Activity Limitations” and asymmetry (X^2^ = 87.00, *p* = 0.01). The distributions of person locations (Fig. 5) indicate that persons with reduced contrast sensitivity (group 2 and 3) and persons with low to moderate asymmetry between the eyes (group 2 and 3) have higher person locations, hence lower VR-QoL, on the “Activity limitations” subscale, compared to the participants with normal contrast sensitivity and no asymmetry between the eyes. The same patterns are evident for the “Symptoms” subscale, however, the participants in group 2 and 3 have person locations closer to the mean of group 1, indicating less correlation between visual function and subjective symptoms.

### Responsiveness

**Fig. 6:**
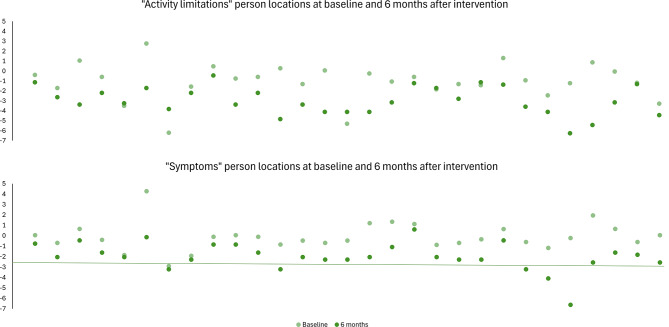
Responsiveness of KORQ-NO. *Note:* Each pair of light and dark green dots represents one participant. The figure illustrates improvements in person locations in both subscales 6 months after intervention (reduced person location indicates better functioning/less symptoms) for most participants. The mean person locations improved significantly with intervention

The Exact Wilcoxon Signed-rank test between person locations at baseline and six months after intervention show statistically significant z-values for both subscales, z = -3.903 (*p* < 0.001) and z = -4.703 (*p* < 0.001), respectively, indicating that KORQ-NO can detect change over time (Fig. 6). NEI VFQ-25 had comparable responsiveness (z = -4.42, *p* < 0.001).

## Discussion

The purpose of this study was to translate and adapt KORQ to the Norwegian language, culture, and environment, and to validate KORQ-NO in a Norwegian keratoconus population. The overall results suggest successful translation and cross-cultural adaptation for use in Norway. The content validity of KORQ-NO was confirmed by the satisfaction of participants and experienced optometrists regarding its relevance and comprehensiveness, with few remarks about the content. The Rasch analysis confirmed acceptable psychometric properties of KORQ-NO, resembling the original KORQ [15], when assessed without the extra item AL3b *“driving in poor visibility”*.

Reliability and responsiveness are crucial attributes for efficient documentation of improvements, relevant in research, clinical practice, and insurance benefit applications to the Norwegian Labour and Welfare Administration [42]. The excellent responsiveness of KORQ-NO confirms the ability to detect change with intervention. To our knowledge, no other validation study has evaluated responsiveness of KORQ, but a clinical study using KORQ before and after corneal collagen crosslinking affirmed this attribute [19]. The person separation indexes suggest higher reliability for “Activity limitations” than “Symptoms”, corresponding with results from other studies [15–19, 21]. Accordingly, the test-retest reliability of “Activity limitations” was superior of “Symptoms”. Measurement errors, more evident in the “Symptoms” subscale, may be explained by several factors. First, scale recalibration, change in persons’ understanding of the scale, is a common bias in prospective measurements [43]. Second, poor face validity with lack of instructions, leading to participant uncertainty in responding to items, may undermine the test-retest reliability [22]. Third, day-to-day fluctuations common in keratoconus potentially add noise and reduce test-retest reliability [7]. However, a sample size of seventeen participants is too low to make conclusions. Previously, two validation studies suggested excellent test-retest reliability, as measured by Spearman’s correlation coefficients [19, 21], and a third study with larger sample size and less measurement errors confirmed good test-retest reliability for both subscales of KORQ [17].

Clinical measurements are objective assessments of visual status and response to correction or treatment, whereas PROMs reflect the subjective experience of the visual impairment [44]. In the current study, lack of statistically significant correlations between clinical measurements and KORQ-NO might be explained by low sample size, as very few participants presented with impaired corrected visual acuity. Nonetheless, there was a statistically significant difference in person locations on “Activity limitations” among groups with different asymmetry between eyes. The graphical displays indicate tendencies of associations with contrast sensitivity as well. Margolis et al. found weak correlations with visual acuity in most vision-specific PROMs, indicating that subjective measures provide complementary insights [44]. These findings are in accordance with studies on keratoconus and associations between VR-QoL and various clinical measurements [6, 10–12, 45]. Future investigation with larger sample size is needed for proper assessment of correlations. Other validation studies have shown weak to moderate correlations between clinical measurements and KORQ [17, 18, 20–22]. Nevertheless, the results emphasize the usefulness of KORQ-NO, as it adds important information on how the patient perceive visual function and symptoms [44].

The results suggest superior targeting of KORQ-NO over NEI VFQ-25, affirming higher reliability of KORQ-NO. This supports the use of KORQ-NO in clinical practice and future studies to complement objective measurements of persons with keratoconus, in line with findings that disease-specific PROMs are usually recommended over generic PROMs due to superior sensitivity and validity [8, 14]. The psychometric properties of the vision-generic NEI VFQ-25 were questioned by Pesudovs et al. [46], congruent with findings in the current study. Regardless, NEI VFQ-25 has been widely used in studies on keratoconus and VR-QoL [6, 10–12, 47], as no keratoconus-specific PROM was available prior to the development of KORQ [15]. Consequently, NEI VFQ-25 is the most relevant PROM to use for construct validity, which was confirmed by the strong negative correlations between NEI VFQ-25 and KORQ-NO. Additionally, the results suggest that the conversion spreadsheets provided by Khadka et al. [15] may be used in the Norwegian population.

The strengths of the current study include the use of Rasch analysis to evaluate the psychometric properties of KORQ-NO and NEI VFQ-25, and evaluation of test-retest reliability, content and construct validity, and responsiveness. The main limitations are low sample sizes in the retest and clinical groups, as well as the main group for Rasch analysis. Sample size was too low for robust evaluations of DIFs and rating scale [38, 48]. Additionally, unidimensionality is affected by sample size, and accurate item fit requires a sample size above 200 [49]. However, 11 participants are sufficient for ICC indications of good test-retest reliability [50], and Rasch analysis with samples 100 may be sufficient when treated with caution and in interaction with qualitative evaluation of the items [39]. Data collection through “Nettskjema” was necessary for feasibility of the study but adds limitations. Potential participants inactive on the “Keratoconus Norway” social media platform was excluded, no information regarding the severity of keratoconus was collected, and collecting retest data over the telephone might add uncertainty to the results. Despite this, the results support the use of KORQ-NO. There is a potential for item reduction without compromising content validity, and future work should aim to reduce the number of items and validate the questionnaire with larger sample size, with particular attention to potential DIFs.

The requirements for Rasch analysis and weaknesses of KORQ-NO need to be addressed further, as neither subscale exhibit perfect unidimensionality. However, the results are comparable to findings in other validation studies of KORQ [15, 16, 18, 20–22]. Although the proportion of statistically significant *t-*tests remained higher than ideal in “Activity limitations” after removal of AL3b, the eigenvalue of the first contrast was lower than the original KORQ [15] and equal to the Australian validation [16]. Deletion of items AL3 *“driving at night”*, AL12 *“doing your hobbies”*, and AL15 *“doing household tasks”* improved dimensionality. DIFs also need to be addressed in the future. With low sample size, DIFs may be results by chance [38]. Ideally, validation with higher sample size should be conducted to confirm or disconfirm the present DIFs [48]. Finally, the disordered response category thresholds of item AL15 *“doing household tasks”* may also be affected by low sample size [38]. However, most participants responded “not at all” to the item, and the retest participants deemed it irrelevant to their condition, justifying the deletion of item AL15 without compromising content validity.

While statistical findings justify removal of AL15, it remains important to consider potential reasons why participants found this item irrelevant beyond sample size limitations. This highlights the broader challenges of cross-cultural adaptation in PROM validation, which involve both methodological and contextual factors. Future research should explore whether discrepancy in item response is due to differences in societal norms, independence in daily living, or varying expectations regarding functional limitations associated with vision impairment. Although there is no global consensus on cross-cultural adaptation of PROMs [26], and a multistep methodology described in literature was followed [25, 26, 30, 31], the process includes strengths and limitations. First, three concurrent, independent translations and the consensus discussion of the translations represents a strength of the translation process. The availability of only one native English-speaking translator within the institution resulted in a single back-translation, which might be perceived as a limitation. However, according to Epstein et al. [26], back translation should not be mandatory. Ideally, the cognitive debriefing should have been more comprehensive, with inclusion of more persons representing the target population [31]. For practical reasons, only one native Norwegian-speaking lay person assessed the questionnaire, which increases risk of persons misunderstanding of items [31]. However, this risk is considered small, as none of the retest participants responding to the questionnaire over the phone expressed uncertainty about the meaning of the items. Further, low numbers of missing data supports low levels of misunderstanding [31].

## Conclusion

Successful translation and adaptation of KORQ to Norwegian language, culture and environment was confirmed by acceptable psychometric properties via Rasch analysis, and evaluations of validity, reliability, and responsiveness. While there is potential for improvements through item reduction of KORQ-NO, the authors support the use of KORQ-NO in research, clinical practice, and as documentation for allowance applications.

## Electronic supplementary material

Below is the link to the electronic supplementary material.

Supplementary Material 1

Supplementary Material 2

Supplementary Material 3

## Data Availability

The dataset used and analyed during the study are available from the corresponding author on reasonable request. According to the University of South-Eastern Norway Open Access policy, the data will be available in Figshare (https://usn.fishare.com) once the article has been published.
