# Supplementary material for: Translation, cross-cultural adaptation, and validation of the Norwegian version of the Keratoconus Outcomes Research Questionnaire
Source: J Patient Rep Outcomes. 2025 May 22;9:57. doi: 10.1186/s41687-025-00896-z (PMC12098238; doi:10.1186/s41687-025-00896-z)
Supplement: Supplementary file 1 — Supplementary Material 1 [file 41687_2025_896_MOESM1_ESM.pdf]

APPENDIX 1
Keratoconus Outcomes Research Questionnaire (KORQ)

**DEMOGRAPHIC DETAILS - please print or circle appropriate options**				
**Title *(please circle)*:**	Mr / Ms / Mrs / Miss / Dr		Date:	
**Surname:**				
**First & Second Names:**				
**Address:**				
			Postcode:	
**Telephone Number:**	Home:	Work:	Mobile:	
**Date of Birth:**			Sex: Male / Female	
**Are you diagnosed with keratocous?**	Yes	No		
**Age at diagnosis:**		yrs / or	Year of diagnosis:	
**Eye involved**	Right Eye	Left Eye	Both Eyes	
**Are you using?**	Glasses	Soft contact lens	Rigid contact lens (RGP)	None
**Any treatment for keratoconus?**	Corneal Crosslinking	Corneal Transplant surgery		None
	others *(Please specify)*…………………………………………			
**Your vision (Visual acuity) with glasses/contact lenses *(If known only)***	Right Eye		Left Eye	
**Your current glasses or contact lens prescription *(If Known only)***	Right Eye		Left Eye	
**Other eye disease/s: *(If known only)***				

Keratoconus Outcomes Research Questionnaire (KORQ)

VISUAL ABILITY

Please select the category that indicates how you think your vision affects your ability to do these activities.

1. How much does your vision interfere with using a computer screen?

Not applicable

Not at all a little quite a bit a lot

2. How much does your vision interfere with driving during the day?

Not applicable

Not at all a little quite a bit a lot

3. How much does your vision interfere with driving during the night?

Not applicable

Not at all a little quite a bit a lot

4. How much does your vision interfere with reading street signs?

Not applicable

Not at all a little quite a bit a lot

5. How much does your vision interfere with watching TV?

Not applicable

Not at all a little quite a bit a lot

6. How much does your vision interfere with walking up/down steps?

Not applicable

Not at all a little quite a bit a lot

7. How much does your vision interfere with avoiding objects in your path?

Not applicable

Not at all a little quite a bit a lot

8. How much does your vision interfere with your ability to do your job?

Not applicable

Not at all a little quite a bit a lot

9. How much does your vision interfere with seeing in the distance?

Not applicable

Not at all a little quite a bit a lot

10. How much do on coming lights interfere with your ability to see, to do your tasks?

Not applicable

Not at all a little quite a bit a lot

11. How much does your vision interfere with doing fine tasks at near?

Not applicable

Not at all a little quite a bit a lot

12. How much does your vision interfere with doing your hobby?

Not applicable

Not at all a little quite a bit a lot

13. How much does your vision interfere with recognizing faces?

Not applicable

Not at all a little quite a bit a lot

14. How much does your vision interfere with seeing in poor light?

Not applicable

Not at all a little quite a bit a lot

15. How much does your vision interfere with doing household tasks? (e.g. cleaning, ironing, washing, washing up)

Not applicable

Not at all a little quite a bit a lot

16. How much does your vision interfere with judging depth?

Not applicable

Not at all a little quite a bit a lot

17. How much does your vision interfere with seeing small objects in the distance? (e.g. golf ball, darts)

Not applicable

Not at all a little quite a bit a lot

18. How much does your vision interfere with sighting tasks? (e.g. camera, microscope, binoculars etc.)

Not applicable

Not at all   a little   quite a bit   a lot

SYMPTOMS

1. How much are you troubled by distorted vision?

Not applicable

Not at all   a little   quite a bit   a lot

2. How much are you troubled by glare and wearing sunglasses all the time?

Not applicable

Not at all   a little   quite a bit   a lot

3. How much does a bright sunny day interfere with your ability to see, to do your tasks?

Not applicable

Not at all   a little   quite a bit   a lot

4. How much are you troubled by wearing rigid gas permeable contact lenses?

Not applicable

Not at all   a little   quite a bit   a lot

5. How much are you troubled by headaches when wearing your glasses/contact lenses?

Not applicable

Not at all a little quite a bit a lot

6. How much are you troubled by dry eyes?

Not applicable

Not at all a little quite a bit a lot

With respect to yours eyes and vision:
7. How much are you troubled by windy days?

Not applicable

Not at all a little quite a bit a lot

With respect to your eyes and vision:
8. How much are you troubled when you are tired?

Not applicable

Not at all a little quite a bit a lot

With respect to your eyes and vision:
9. How much are you troubled by dry days?

Not applicable

Not at all a little quite a bit a lot

With respect to your eyes and vision:
10. How much are you troubled by dusty days?

Not applicable

Not at all   a little   quite a bit   a lot

With respect to your eyes and vision:
11. How much are you troubled by smoky environments?

Not applicable

Not at all   a little   quite a bit   a lot

Thank you!
