# Supplementary material for: Translation, cross-cultural adaptation, and validation of the Norwegian version of the Keratoconus Outcomes Research Questionnaire
Source: J Patient Rep Outcomes. 2025 May 22;9:57. doi: 10.1186/s41687-025-00896-z (PMC12098238; doi:10.1186/s41687-025-00896-z)
Supplement: Supplementary file 2 — Supplementary Material 2 [file 41687_2025_896_MOESM2_ESM.docx]

***Appendix 2***

***Reliability and validity according to the COSMIN checklist***

#### Reliability; Internal consistency, test-retest reliability, and measurement error

COSMIN defines internal consistency as “interrelatedness among items” (1), meaning that all items in a questionnaire contribute to measure the same latent trait. Several components of the Rasch analysis contribute to the evaluation of internal consistency, including assessment of unidimensionality, PSI, and response dependency (2).

The assessment of test-retest reliability is important to confirm whether the results of a PROM are stable when administered to the same person more than once without change in the underlying condition (3). The Intraclass Correlation Coefficient (ICC), 2-way mixed-effects model with absolute agreement (4), was calculated for the person locations for both subscales from initial responses collected from “Nettskjema” and responses collected over the telephone three to four weeks later. A sample size of 11 participants is sufficient to calculate ICC to show reliability above 0.7 when anticipated reliability is 0.9 (5). Bland-Altman plots with Limits of Agreement complement the ICCs.

#### Content and construct validity

Content validity is defined as “the degree to which the content of a PROM is an adequate reflection of the construct to be measured” (1). Qualitative judgement of the relevance and comprehensiveness of the items of KORQ-NO were made by the participants in the retest group and three optometrists experienced with patients with keratoconus. The participants were asked to identify irrelevant items in KORQ-NO and critical issues not covered by the items.

Construct validity is defined as “the degree to which the scores of a PROM are consistent with hypotheses based on the assumption that the PROM validly measures the construct to be measured” (1). COSMIN refers to the following three aspects of construct validity:

Structural validity, defined as “the extent to which items, rating scale categories, and people cohere to form a measure that substantively reflects the requirements of the Rasch model” (6), is confirmed by fulfilment of key requirements of the Rasch model described above.

Hypothesis testing to confirm construct validity, was done by comparing KORQ-NO with NEI VFQ-25. There is no gold standard PROM for the assessment for VR-QoL, and NEI VFQ-25 was chosen as it is widely used instrument for the assessment of VR-QoL in keratoconus (7). NEI VFQ-25 consists of twenty-five items forming eleven subscales. The rating scale varies across items, and a conversion table guides conversion of the numeric values and summation to a composite score between 0 and 100. Higher scores relate to higher VR-QoL and functioning (8). Spearman correlations were calculated to assess correlations between the person locations for both subscales of KORQ-NO and the composite score of NEI VFQ-25, with the hypothesis of strong negative correlations. Correlations were interpreted as zero (r_s_ = 0.0), weak (r_s_ = 0.1-0.3), moderate (r_s_ = 0.4-0.6), strong (r_s_ = 0.7-0.9) or perfect (r_s_ = 1.0), with significance level set to p<0.05 (9). Additionally, Rasch analysis were run to compare the psychometric properties of NEI VFQ-25 to KORQ-NO.

Additionally, data from the clinical study were analysed, including habitual binocular distance and near visual acuity (VA in logMAR), contrast sensitivity and asymmetry in distance VA between the two eyes. Spearman correlations between person locations for both subscales and the four clinical measurements were calculated. Further, participants were divided into groups based on visual functioning (table 1). Person locations between groups were compared by chi square statistics and presented graphically.

***Table 1***

*Categorization of visual functions*

|  | **Group1** | **Group 2** | **Group 3** | **Group 4** |
| --- | --- | --- | --- | --- |
| Visual acuity  (distance and near) | No visual impairment | Mild visual impairment | Moderate visual impairment | Blindness |
| logMAR | <0.00-0.28 | 0.30-0.48 | 0.50-1.28 | >1.30 |
| Contrast sensitivity | Normal | Normal Age 60+ | Moderate reduction | Severe reduction |
| MARS CS | 1.92-1.72 | 1.67-1.52 | 1.48-1.04 | 0.52-1.00 |
| Asymmetry | No asymmetry | Mild asymmetry | Moderate symmetry | Severe asymmetry |
| # lines difference in distance VA | <1 | 1-2 | 2-3 | >3 |

*Note: Categorization of visual acuity and contrast sensitivity in accordance with the International Classification of Diseases, Eleventh Revision (ICD-11)* (10) *and the MARS CS User Manual* (11)*.*

Cross-cultural validity involves testing of the hypothesis that all items work equally between various groups (3), for example across groups of different gender, age or nationalities, referred to as invariance above. To complement the DIF analysis, Spearman correlations between the RUMM2030+ calculated person locations and the person locations found by using the ready-made conversion spreadsheets provided by Khadka et al. (12) (available at <http://links.lww.com/OPX/A287> and <http://links.lww.com/OPX/A288>) were assessed to evaluate whether the ready-made conversion spreadsheets were appropriate for a Norwegian keratoconus population.

#### Responsiveness

Responsiveness is defined as “the ability of a PROM to detect change over time in the construct to be measured” (1), highly relevant in studies involving clinical interventions. Data from a clinical intervention study was used to assess the responsiveness of KORQ-NO. Stable estimates of responsiveness require a sample size of at least 50 (5). Due to the limited sample size available, the Exact Wilcoxon Signed-rank test (2-tailed sign. level p<0.001) was used to compare person locations for “Activity limitations” and “Symptoms” at baseline and six months after the intervention of scleral lens fitting.(13). The responsiveness of composite scores of NEI VFQ-25 was calculated for additional comparison of the questionnaires.

# References

1. Mokkink LB, Terwee CB, Knol DL, Stratford PW, Alonso J, Patrick DL, et al. The COSMIN checklist for evaluating the methodological quality of studies on measurement properties: A clarification of its content. BMC Med Res Methodol. 2010 Mar 18;10(1):22.

2. Andrich D, Marais I. A Course in Rasch Measurement Theory: Measuring in the Educational, Social and Health Sciences [Internet]. Singapore: Springer Nature Singapore; 2019 [cited 2024 Jan 30]. (Springer Texts in Education). Available from: https://link.springer.com/10.1007/978-981-13-7496-8

3. Mokkink LB, Prinsen CA, Patrick DL, Alonso J, Bouter LM, de Vet HC, et al. COSMIN Study Design checklist for Patient-reported outcome measurement instruments. COSMIN [Internet]. 2019; Available from: https://www.cosmin.nl/wp-content/uploads/COSMIN-study-designing-checklist_final.pdf

4. Koo TK, Li MY. A Guideline of Selecting and Reporting Intraclass Correlation Coefficients for Reliability Research. J Chiropr Med. 2016 Jun 1;15(2):155–63.

5. Fayers PM, Machin D. Quality of Life: The assessment, analysis and reporting of patient-reported outcomes. 3rd edition. West Sussex, UK: Wiley Blackwell; 2016. 648 p.

6. Winckel AV de, Kozlowski AJ, Johnston MV, Weaver J, Grampurohit N, Terhorst L, et al. Reporting Guideline for RULER: Rasch Reporting Guideline for Rehabilitation Research: Explanation and Elaboration. Arch Phys Med Rehabil. 2022 Jul 1;103(7):1487–98.

7. Margolis MK, Coyne K, Kennedy-Martin T, Baker T, Schein O, Revicki DA. Vision-Specific Instruments for the Assessment of Health-Related Quality of Life and Visual Functioning. PharmacoEconomics. 2002 Oct 1;20(12):791–812.

8. Mangione CM, Lee PP, Gutierrez PR, Spritzer K, Berry S, Hays RD. Development of the 25-list-item National Eye Institute Visual Function Questionnaire. JAMA Ophthalmol. 2001;119(7):1050–8.

9. Akoglu H. User’s guide to correlation coefficients. Turk J Emerg Med. 2018 Aug 7;18(3):91–3.

10. World Health Organization. ICD-11: International classification of diesases (11th revision) [Internet]. 2022 [cited 2024 Jun 7]. Available from: https://icd.who.int/en

11. Mars Perceptrix Corporation. The Mars Numeral Contrast Sensitivity Test user manual [Internet]. 2012 [cited 2024 Aug 22]. Available from: https://marsperceptrix.com/sites/default/files/downloads/MarsNumeralCSTestUserManualEnglish.pdf

12. Khadka J, Schoneveld PG, Pesudovs K. Development of a Keratoconus-Specific Questionnaire Using Rasch Analysis. Optom Vis Sci. 2017 Mar;94(3):395.

13. Marshall E, Marquier B. Wilcoxon Signed-rank test in SPSS [Internet]. Statstutor Community Project; 2024. Available from: https://www.google.com/url?sa=t&rct=j&q=&esrc=s&source=web&cd=&ved=2ahUKEwin6fCgz_uGAxUPIRAIHRokD9YQFnoECCsQAQ&url=https%3A%2F%2Fwww.sheffield.ac.uk%2Fmedia%2F35102%2Fdownload%3Fattachment&usg=AOvVaw3JSndktor4dlHq90A1rj-4&opi=89978449
