# Supplementary material for: Translation, cross-cultural adaptation, and validation of the Norwegian version of the Keratoconus Outcomes Research Questionnaire
Source: J Patient Rep Outcomes. 2025 May 22;9:57. doi: 10.1186/s41687-025-00896-z (PMC12098238; doi:10.1186/s41687-025-00896-z)
Supplement: Supplementary file 3 — Supplementary Material 3 [file 41687_2025_896_MOESM3_ESM.pdf]

		Response categories				
		1	2	3	4	NA
Item	Statement	n	n	n	n	n
	**How much does your vision interfere with**					
AL1	using digital devices	35	44	66	17	3
AL2	driving during the day	58	49	39	8	11
AL3	driving during the night	13	28	51	62	11
AL3b	driving during bad weather conditions	15	29	55	53	13
AL4	reading signs	18	46	56	39	6
AL5	watching TV	29	52	62	18	4
AL6	walking in stairs	70	46	32	14	3
AL7	walking onto things	87	43	29	2	4
AL8	your ability to do your job	47	46	47	12	13
AL9	seeing in the distance	12	33	61	55	4
AL10	oncoming lights	11	33	57	59	8
AL11	doing fine tasks at near	23	58	61	19	4
AL12	doing your hobby	39	54	50	15	7
AL13	recognizing faces	42	35	56	29	3
Al14	seeing in poor light	10	30	60	60	5
AL15	doing household tasks	93	29	37	2	4
AL16	judging depth	26	48	52	36	3
AL17	seeing small objects in distance	17	35	49	54	10
AL18	sighting tasks	11	40	48	40	26
	**How much are you troubled by**					
SYM1	distorted vision	23	49	68	23	2
SYM2	glare and wearing sunglasses all the time	23	45	59	37	1
SYM3	bright sunny days	44	62	47	11	1
SYM4	wearing rigid gas permeable contact lens	14	26	36	37	52
SYM5	headaches when wearing your glasses/cc	35	42	54	24	10
SYM6	dry eyes	15	40	58	51	1
SYM7	lots of wind	22	49	61	32	1
SYM8	when you are tired	8	27	61	68	1
SYM9	dry air	14	42	62	45	2
SYM10	dusty air	11	30	65	56	3
SYM11	smokey air	18	53	52	30	12
